# Seroprevalence of Antibodies to Avian Influenza Virus A (H5N1) among Residents of Villages with Human Cases, Thailand, 2005^1^

**DOI:** 10.3201/eid1505.080316

**Published:** 2009-05

**Authors:** Rapeepan Dejpichai, Yongjua Laosiritaworn, Pilaipan Phuthavathana, Timothy M. Uyeki, Michael O’Reilly, Nattaphon Yampikulsakul, Sumreung Phurahong, Phisanu Poorak, Jarunee Prasertsopon, Rumporn Kularb, Kannika Nateerom, Narumol Sawanpanyalert, Chuleeporn Jiraphongsa

**Affiliations:** Thai Ministry of Public Health, Nonthaburi, Thailand (R. Dejpichai, Y. Laosiritaworn, M. O’Reilly, N. Yampikulsakul, S. Phurahong, N. Sawanpanyalert, C. Jiraphongsa); Mahidol University, Bangkok, Thailand (P. Phuthavathana, P. Poorak, J. Prasertopon, R. Kularb, K. Nateerom; Centers for Disease Control and Prevention, Atlanta, GA, USA (T.M. Uyeki)

**Keywords:** Influenza, respiratory infections, seroprevalence, H5N1, antibodies, humans, Thailand, research

## Abstract

In 2005, we assessed the seroprevalence of neutralizing antibodies to avian influenza virus A (H5N1) among 901 residents of 4 villages in Thailand where at least 1 confirmed human case of influenza (H5N1) had occurred during 2004. Although 68.1% of survey participants (median age 40 years) were exposed to backyard poultry and 25.7% were exposed to sick or dead chickens, all participants were seronegative for influenza virus (H5N1).

## Abstract 2

No evidence of influenza virus A (H5N1) neutralizing antibodies was found in residents of 4 villages where human cases had occurred the previous year.

Three apparent waves of highly pathogenic avian influenza virus A (H5N1) infection in humans occurred in Thailand from early 2004 through 2006; these waves, which corresponded to influenza (H5N1) outbreaks in poultry, resulted in 25 confirmed human cases and 17 deaths (*1*–*4*). However, the frequency of asymptomatic and clinically mild cases of influenza (H5N1) infection was unknown in areas where these outbreaks occurred. In 2005, we conducted a cross-sectional seroprevalence study of influenza virus (H5N1) antibodies among residents of 4 rural villages in Thailand where at least 1 human influenza (H5N1) case had occurred in 2004. Backyard poultry farming is common in these villages, but the villages have no live poultry markets.

## Methods

The study was conducted during October 11–27, 2005, among residents of 4 rural villages in central and northern Thailand where influenza (H5N1) outbreaks in poultry and human influenza (H5N1) cases had occurred: village A in Prachin Buri Province (1 confirmed case), village B in Kamphaeng Phet Province (1 probable case, 1 confirmed case), village C in Sukhothai Province (1 confirmed case), and village D in Phetchabun Province (1 confirmed case) (Figure). Illness onset in these case-patients occurred from August 31 through October 8, 2004. Residents of any of the villages for at least 2 weeks before and after illness onset of the respective case-patient person in each village were eligible to participate in the study. Participants were enrolled by random selection from lists of village residents or by convenience sampling. Village residents were excluded if they had influenza (H5N1) diagnosed from August 17 through October 22, 2004, were <18 years of age and did not have parental consent, had an underlying coagulopathy, or were taking anticoagulant drugs within 2 weeks of enrollment. Written informed consent was obtained from all study participants or their proxies.

**Figure:**
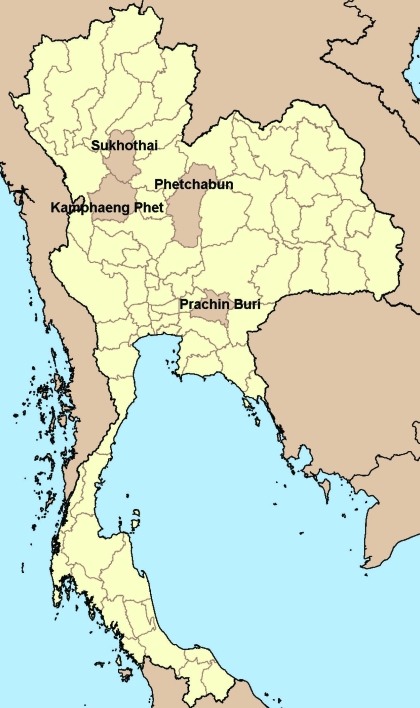
Province location of study villages with laboratory-confirmed avian influenza A (H5N1) cases in humans, Thailand, 2004. (Adapted from http://commons.wikimedia.org/wiki/Image:BlankMap_Thailand.png)

Using a standard questionnaire, trained interviewers collected demographic and exposure data through brief face-to-face interviews with study participants. Exposure was defined as either direct contact (touching) or close contact (within 1 m without direct contact) with chickens or other poultry or with a person with confirmed influenza (H5N1) infection. A 5-mL blood specimen was collected from participants >5 years of age, and a 3-mL specimen was collected from those <5 years of age. Serum samples were separated at a local hospital and transported on wet ice to a laboratory within 48 h after collection. Serologic testing by microneutralization (MN) assay was performed in an enhanced biosafety level-3 containment facility in accordance with a slightly modified version of a protocol described previously (*5*–*7*). Influenza virus A/Thailand/1(KAN-1)/2004 (H5N1) was selected for the MN assay because of its antigenic similarity to influenza virus (H5N1) isolates from humans in Thailand (*2*). Immunofluorescence with use of 293T cells transfected with hemagglutinin H5N1 recombinant plasmid as the test antigen was used to confirm MN assay results. In accordance with our modified protocol, we considered an influenza virus (H5N1) neutralizing antibody titer >40 (equivalent to >80 in other protocols) to be a positive result (*5*–*7*).

Epi Info 2002 (Centers for Disease Control and Prevention, Atlanta, GA, USA) was used to enter and analyze study data. Mean, median, and proportion values were calculated for variables and compared by using bivariate analysis. The χ^2^ test was used for most analyses, analysis of variance was used to compare means from the convenience sample with those from the random sample, and the Fisher exact test was used if expected cell values were <5. Differences between the 2 sample groups were considered significant at p<0.05. The study was approved by the Ethical Review Committee for Research in Human Subjects, Thai Ministry of Public Health.

## Results

The study population consisted of 901 participants: 228 from village A (28.1% of village residents), 203 from village B (28.4%), 209 from village C (30.5%), and 260 from village D (19.6%). Their median age was 40 years (range 2–101 years), and 42.4% were male. The 901 participants were enrolled in 2 ways: 131 (14.5%) by random selection (out of 838 randomly selected villagers: 15.6% participation), and 770 (85.5%) by convenience sampling. The 2 groups of study participants did not differ significantly by demographic characteristics, history of illness, or exposure to poultry (Table 1). Most participants (68.1%) reported direct or close contact with backyard poultry, 25.7% reported direct or close contact with sick or dead chickens, and 7.1% reported close contact with a person with confirmed influenza (H5N1) infection (Table 1). Of 110 participants who reported a history of acute respiratory symptoms, 74.5% reported direct or close contact with backyard poultry, 31.8% reported direct or close contact with sick or dead chickens, and 13.6% reported close contact with a person with confirmed influenza (H5N1) infection (data not shown). All participants were seronegative for influenza virus (H5N1) neutralizing antibodies (Table 2).

**Table 1:** Characteristics of the study population, overall and by method of selection, Thailand, 2005

Characteristic	Random sample (n = 131), no. (%)	Convenience sample (n = 770), no. (%)	p value	Total sample (N = 901), no. (%)
Age group, y			0.30	
1–14	19 (14.5)	178 (23.1)		197 (21.9)
15–29	14 (10.7)	99 (12.9)		113 (12.5)
30–44	37 (28.2)	177 (23.0)		214 (23.8)
45–59	32 (24.4)	175 (22.7)		207 (22.9)
60–74	24 (18.3)	109 (14.2)		133 (14.8)
75–89	4 (3.1)	28 (3.6)		32 (3.6)
90–104	1 (0.8)	4 (0.5)		5 (0.5)
Occupation			0.04	
Plant farmer	59 (45.0)	236 (30.6)		295 (32.7)
Animal farmer	3 (2.3)	11 (1.4)		14 (1.6)
Farmer (plant and animal)	2 (1.5)	7 (1.0)		9 (0.10)
Merchant	2 (1.5)	13 (1.7)		15 (1.7)
Government officer	0	3 (0.4)		3 (0.4)
Other (employee, housekeeper)	42 (32.1)	346 (44.9)		388 (43.0)
Missing	23 (17.6)	154 (20.0)		177 (19.6)
Sex			0.70	
Male	58 (44.3)	324 (42.1)		382 (42.4)
Female	73 (55.7)	446 (57.9)		519 (57.6)
Risk factors			0.60	
Direct or close contact with backyard poultry (including chickens)	89 (67.9)	525 (68.2)		614 (68.1)
Direct or close contact with backyard chickens	86 (65.6)	519 (67.4)		605 (67.1)
Direct or close contact with dead/sick chicken	36 (27.5)	196 (25.5)		232 (25.7)
Close contact with a person with a confirmed case of avian influenza A	13 (9.9)	51 (6.6)		64 (7.1)
Acute respiratory symptoms*			0.31	
Symptoms	12 (9.2)	98 (12.7)		110 (12.2)
No symptoms	119 (90.8)	672 (87.3)		791 (87.89)
Influenza-like illness†			0.39	
Symptoms	7 (5.3)	61 (7.9)		68 (7.5)
No symptoms	124 (94.7)	709 (92.1)		833 (92.5)

*Acute respiratory symptoms were rhinorrhea, cough, sore throat, or dyspnea. †Influenza-like illness was defined as a temperature >38.0°C in conjunction with any of the following: rhinorrhea, cough, sore throat, or dyspnea.

**Table 2:** Avian influenza virus A (H5N1) neutralizing antibody titers among study participants (N = 901), as determined by microneutralization assay, Thailand, 2005

Village	No. residents	No. residents by antibody titer						
		<5	5	10	20	40	80	>80
A	228	227	1	0	0	0	0	0
B	204	202	2	0	0	0	0	0
C	209	202	6	0	1*	0	0	0
D	260	257	2	1†	0	0	0	0
Total	901	888	11	1	1	0	0	0

*Serum obtained from a 52-year-old woman (farmer) in village C without history of respiratory symptoms who reported contact with a sick/dead chicken and live poultry. †Serum obtained from an 18-year-old man in village D without history of respiratory symptoms who reported contact with a sick/dead chicken and live poultry.

## Discussion

Participants in this study were from villages in central and northern Thailand where widespread, confirmed outbreaks of influenza (H5N1) infection in poultry and at least 1 human influenza (H5N1) case had occurred during 2004. A substantial proportion of participants reported exposure to backyard poultry, including contact with sick or dead chickens, the primary risk factor for influenza (H5N1) infection (*8*,*9*). Nevertheless, we found no serologic evidence of mild or subclinical influenza (H5N1) infection, suggesting that clade 1 influenza virus A (H5N1) strains circulating in Thailand among backyard poultry during 2004 did not transmit easily to our study population.

Our findings differ from those from a study of poultry workers in Hong Kong, among whom the estimated seroprevalence of influenza virus (H5N1) neutralizing antibodies was 10% during the 1997 outbreak (*10*). The Hong Kong poultry workers, however, likely had much greater intensity of exposure to poultry infected with influenza virus (H5N1) than our study population had. Furthermore, the clade 1 influenza virus (H5N1) strains that infected poultry and humans in Thailand during 2004 were antigenically and genetically distinct from the clade 0 influenza virus (H5N1) strains that caused the 1997 outbreak in Hong Kong (*11*). Our finding of no serologic evidence of asymptomatic or mild influenza (H5N1) infection among Thai villagers is consistent with findings from smaller influenza virus (H5N1) seroprevalence studies among workers in live poultry markets in the People’s Republic of China (*12*), among villagers exposed to backyard poultry infected with clade 1 influenza virus (H5N1) in rural Cambodia (*13*), among poultry workers exposed to poultry infected with clade 2.2 influenza virus (H5N1) in northern Nigeria (*14*), and among poultry farmers exposed to poultry infected with clade 1 influenza virus (H5N1) in Thailand (*7*). Results of studies conducted since 2004 thus suggest that the risk for influenza (H5N1) infection is low among persons exposed to infected poultry; however, our finding of no serologic evidence of asymptomatic or mild influenza (H5N1) infection among Thai villagers suggests that the high case-fatality proportion in Thailand (17 deaths among 25 persons with confirmed infection) may accurately reflect the severity of the infection in Thailand.

Our study had 3 notable limitations. First, because study participants, most of whom were enrolled by convenience sampling, were generally older than the populations of the villages in which they resided (Thai Ministry of Public Health, unpub. data), our findings may not be generalizable to these villages’ populations. Second, because the study was conducted in 2005, some participants may not have accurately recalled relevant exposures or symptoms from 2004 when the influenza (H5N1) cases occurred. Third, some participants with mild or asymptomatic influenza (H5N1) infection in 2004 may not have generated an antibody response strong enough or durable enough to be detected in serum samples collected in October 2005.

Further data are needed on the natural history and kinetics of the immune response to influenza (H5N1) infection over time among severely ill persons who survived, as well as among those with clinically mild illness. Such prospective serial data may help researchers interpret the significance of low levels of influenza virus (H5N1) neutralizing antibody titers, as well as the results of additional seroprevalence studies. In addition, because influenza virus (H5N1) strains continue to evolve, additional seroprevalence studies to estimate human risk for infection are needed worldwide among populations exposed to the virus, including poultry workers, rural residents, market workers, farm workers, healthcare workers, and family members of infected persons.
